# Strong effect of Ecuador’s conditional cash transfer program on childhood mortality from poverty-related diseases: a nationwide analysis

**DOI:** 10.1186/s12889-019-7457-y

**Published:** 2019-08-17

**Authors:** Ana L. Moncayo, Guillermo Granizo, Mario J. Grijalva, Davide Rasella

**Affiliations:** 1 Centro de Investigación para la Salud en América Latina (CISeAL), Facultad de Ciencias Exactas y Naturales, Pontificia Universidad Católica del Ecuador, Apartado: 17-01-2184, Av. 12 de octubre 1076, Quito, Ecuador; 2 Department of Biomedical Sciences, Infectious and Tropical Disease Institute, Heritage College of Osteopathic Medicine, Ohio University, Athens, Ohio USA; 3 Instituto Gonçalo Muniz, Fundação Oswaldo Cruz, Salvador, Bahia Brazil; 4 Instituto de Saúde Coletiva, Universidade Federal da Bahia, Salvador, Bahia Brasil; 5 Department of Primary Care and Public Health, Public Health Policy Evaluation Unit, School of Public Health, Imperial College London, London, UK

**Keywords:** Conditional cash transfer, *Bono de Desarrollo Humano*, Social determinants, Under-5 mortality rates, Ecuador

## Abstract

**Background:**

The mortality rate in children under 5 years old (U5MR) has decreased considerably in Ecuador in the last decade; however, thousands of children continue to die from causes related to poverty. A social program known as *Bono de Desarrollo Humano* (BDH) was created to guarantee a minimum level of consumption for families and to reduce chronic malnutrition and preventable childhood diseases. We sought to evaluate the effect of the BDH program on mortality of children younger than 5 years, particularly from malnutrition, diarrheal diseases, and lower respiratory tract infections.

**Methods:**

Mortality rates and BDH coverage from 2009 to 2014 were evaluated from the 144 (of 222) Ecuadorian counties with intermediate and high quality of vital information. A multivariable regression analyses for panel data was conducted by using a negative binomial regression model with fixed effects, adjusted for all relevant demographic and socioeconomic covariates.

**Results:**

Our research shows that for each 1% increase in BDH county coverage there would be a decrease in U5MR from malnutrition of 3% (RR 0.971, 95% CI 0.953–0.989). An effect of BDH county coverage on mortality resulting from respiratory infections was also observed (RR 0.992, 95% CI 0.984–0.999). The BDH also reduced hospitalization rates in children younger than 5 years, overall and for diarrhea.

**Conclusions:**

A conditional cash transfer program such as BDH could contribute to the reduction of mortality due to causes related to poverty, such as malnutrition and respiratory infections. The coverage should be maintained -or increased in a period of economic crisis- and its implementation strengthened.

**Electronic supplementary material:**

The online version of this article (10.1186/s12889-019-7457-y) contains supplementary material, which is available to authorized users.

## Background

Child survival has substantially improved worldwide in the past 25 years as a result of the efforts to reach the Millennium Development Goal (MDG) 4 of a two-thirds reduction in the under-five mortality rate (U5MR) between 1990 and 2015. Indeed, the global U5MR dropped 56%, from 93 deaths per 1000 live births in 1990 to 41 in 2016 [1]. Latin American and the Caribbean (LAC) have reduced the under-five mortality rate by 67% since 1990.

However, despite this progress, millions of children continue to live and die in conditions that are unacceptable. Globally, it has been described that the majority of child deaths are due to poverty and as a result of diseases that can be prevented and treated easily and economically [2]. To improve the socioeconomic conditions of disadvantaged citizens, social assistance programs have been implemented by many countries around the world. Globally, 77% and 42% of countries have unconditional and conditional cash transfers, respectively; however, significant variations in spending are observed across countries and regions [3].

Conditional Cash Transfer Programs (CCTPs) offer cash benefits to poor families that meet certain conditions associated with human capital development such as children’s school attendance and health checkups. In Ecuador, the largest social assistance program is *Bono de Desarrollo Humano* (BDH) that began operations in 2003, preceded by the unconditional transfer program *Bono Solidario*, which began in 1998 [4]. In 2003, BDH represented 0.49% of Gross Domestic Product (GDP) and from 2007 to 2013 had an upward trend reaching between 0.55 and 0.67%, but then fell to 0.43% of GDP in 2014 and 0.26% in 2015 [5]. In fact, between 2007 and 2013 the usual number of beneficiary families had oscillated between 1 million and 1.2 million and the money was transferred to low-income mothers below the poverty line according to the Social Registry in all counties of the country [4]. Processes of social mobility promoted by the government and the modification of the target population to people living in extreme poverty due to fiscal constraints, caused the number of beneficiaries to decrease by 56.7% between 2013 and 2014 (1,026,114 beneficiary families in 2013 to 444.562 beneficiary families in 2014) [4, 6, 7]. The coverage was reduced because the changes in the eligibility criteria only involved the graduation strategy but not the entry of new potential beneficiaries [5].

Since 2013, BDH provided conditional cash transfers of US$50 per month to target families with members under 18 years old and currently involves an additional transfer based on the number of children for up to a maximum additional US$150 [7]. The required behaviors included both attendance by both the mother and children at preventive health check-ups and requiring a minimum percentage of attendance at school for school-age children. The aim of the program is to guarantee a minimum level of consumption for families and to contribute to the reduction of chronic malnutrition and preventable diseases for children under the age of five [7]. Because of the education condition, there is an expectation that children will have better opportunities later in life [8].

These programs have been shown to have led to a reduction in poverty [9] and to have positive effects on education [10] and health [11–13]. Programs such as *Bolsa Familia* (Brazil) [14] and *Progresa* (Mexico) [15] helped to reduce child mortality and morbidity due to causes associated with poverty, such as malnutrition and diarrhea. However, any country has implemented CCTPs heterogeneously, with different conditions and varying enforcement, with different eligibility rules and varying values in the monetary allowance, and different local factors, which can influence the effectiveness of the program. Therefore, there is an urgent need for country-specific evaluations of CCTs.

### Child mortality in Ecuador

Ecuador is among the 24 (of 81) low- and lower-middle income countries that met MDG 4 [1]. The U5MR declined in the country from 57 deaths per 1000 live births in 1990 to 21 in 2016 with an annual rate of reduction of 3.8% [1]. The poverty reduction and the government investment in health could have a positive impact on reducing U5MR. Between 2001 and 2014, high economic growth and changes in the distribution of income helped lift 1.4 million people out of poverty [5]. Labor income accounted for a decline of 10.7 percentage points in the national poverty and government transfers were responsible for a more than 3 percentage point reduction in total poverty [5]. Improved access to basic services and increased net enrollment rate in education also contributed to improved welfare [5]. The government investment in health increasing from 1.5% of GDP per year in 2007 to 4.5% in 2014 [16] ensuring coexistence of infrastructure, medical supplies and health care providers and reinforcing preventive and primary health care. In addition, since 2007, child development became a political priority with particular emphasis on the strengthening of care services and fighting against malnutrition [6]. BDH should have a positive impact on childhood mortality through poverty reduction, human capital development and health care utilization, which constitute one of the conditionalities of the program. [14, 15]

In Ecuador, some studies have analyzed the role of BDH on child development and nutrition [17–19], but no studies to date have addressed its effect on childhood survival. Therefore, the aim of this study was evaluating the effect of BDH on U5MR in Ecuadorian counties, particularly mortality from poverty-related causes, including diarrhea, malnutrition and low respiratory infections (because are among the leading causes of death in children under 5 years worldwide [1]) and on some of the potential intermediate mechanisms (hospitalization rates).

## Methods

### Study design

We performed a mixed ecological study with the Ecuadorian counties as the units of analysis, during 2009–2014. Previous years could not be included in the study because data on BDH coverage were only available in a public repository since 2009. This ecological design is a combination of an ecological multiple-group and time-trend study design. We analyzed the quality of information on births and deaths for all 222 counties according to a specific criterion [20] and counties were included in the study only if they had intermediate and high quality information for the duration of the study period. This criterion considered five indicators: age-standardized mortality rate; the ratio between registered and estimated birth rates; mean relative deviation of the mortality rate; mean relative deviation of the birth rate; and proportion of poorly defined deaths [20]. We ordered each of the indicators from worst to best in terms of quality and a position was assigned according to the order. The final score was obtained by adding the value of the position for the 5 indicators. Finally we obtained the tertiles of the final score distribution to categorize in low, intermediate and high quality of information.

We defined as dependent variables: a) U5MR (number of deaths of children under 5 years per 1000 live births); b) cause-specific U5MR (number of deaths of children under 5 years resulting from diarrhea diseases, malnutrition or lower respiratory tract infections per 1000 live births); c) under-five hospitalization rate defined as number of children under 5 years who leave a hospital after receiving care due to diarrhea diseases, malnutrition or lower respiratory infections per 1000 live births). We used hospital discharge rates as a *proxy* because rates of admission to hospital were not available. The specific causes of mortality and hospitalization were created according the International Classification of Diseases (ICD), 10th revision: diarrheal diseases (A00, A01, A03, A04, A06-A09); malnutrition (E40-E46); lower respiratory infections (J10-J18, J20-J22); and external causes (V01–99). External causes were included as a control variable because there is not an expected effect of the program due to these causes. All these dependent variables were obtained by direct calculation [14].

In order to evaluate different aspects of effectiveness [14], the primary explanatory variable was BDH coverage and two indicators were created: 1) coverage of the eligible population (EP): number of families enrolled in BDH program in a county / number of eligible families in the same county; 2) coverage of the county population (CP): number of individuals enrolled [obtained by multiplying the number of beneficiary families by the average family size of the county] / total population of the same county). While the first indicator of coverage evaluates the effectiveness of the intervention only on the eligible population, the second is also able to catch the externalities, or spill-over effects, that the money allowances injected – through the beneficiaries – on the overall economy of the community, as shown elsewhere [14]. Results from BDH-EP coverage are very similar (and only slightly lower) with BDH-CP and are shown in the Additional files 1 and 2.

Based on a literature review, we identified a set of covariates as determinants of under-five mortality given their potential to confound the effect on dependent variables. The following covariates were used in the analysis [14, 21, 22]: per capita income, illiteracy, percentage of households with inadequate sanitation, total fertility rate, number of physicians per 10,000 residents and bed rate per 1000 residents.

### Data sources

The data sources for this study were the National Institute of Statistics and Census [23] (Database of births and deaths, Population Census 2001–2010, hospitalizations) and the National System of Information [24] (Integrated System of Knowledge and Social Statistics of Ecuador and Projections and demographic studies). Data were obtained at a county level, which is the lowest level with information about BDH. Except for census years 2001 and 2010, we estimated some covariates by linear interpolation or extrapolation as has been done in previous studies [14, 21, 22, 25].

### Statistical analyses

We measured the effect of BDH coverages on U5MR and hospitalization rate using conditional negative binomial regression models for panel data with fixed-effects specification (counties as units of analysis with observations repeated over time) [14, 26, 27]. Negative binomial regression is used when the outcome to be analyzed is a count or a rate (with an offset variable) with a tendency to over-dispersion [26]. Fixed-effect models include a term to control for unmeasured time-invariant county characteristics (such as geography and cultural practices) and to correct for correlation of repeated measures. To evaluate the association between BDH coverage and U5MR (overall and specific), we calculated mortality rate ratios (95% CI), both crude and adjusted for a set of demographic, social and economic determinants as covariates [14]. A time variable was also included in the models to control for secular trends of mortality. All analyses were performed using STATA version 12.0 software (Stata Corporation, College Station, TX, USA).

## Results

After we applied the criteria for inclusion (vital statistics quality criterion), we selected 144 counties (64.9%) with intermediate and high registration of vital statistics (death and live births) of the 222 Ecuadorian counties. The mean under-5 mortality rate decreased from 15.2 to 12.9 (15.1% reduction) per 1000 live births in the studied counties, during 2009–2014. Among selected causes, there was a large decline in the U5MR attributed to diarrheal diseases (79% reduction) (Table 1). The BDH coverage showed a progressive decrease from 2009 to 2014 (CP: 62.5%; EP: 63.8%). In terms of absolute number, this meant a reduction from 1,066,892 families covered by BDH in 2009 to 348,404 families covered in 2014. Although, the overall under-5 hospitalization rate increased slightly during the study period, the rate by diarrheal diseases and malnutrition decreased by 1.9% and 27.6%, respectively. We observed marked improvements in socioeconomic conditions during the study period. Per capita income increased by 42.1%, the percentage of households with inadequate sanitation decreased by 5.7% and the proportion of illiterate individuals decreased by 11.6%. There were also reductions in the total fertility rate (12.0%) (Table 1).

**Table 1:** Descriptive analysis of study variables (*N* = 144), 2009–2014, Ecuador

	2009	2010	2011	2012	2013	2014	Percentage change 2009–14
Mortality rate for children younger than 5 years (per 1000 livebirths)							
Overall	15.2 (7.5)	15.4 (9.8)	14.8 (9.0)	13.0 (7.5)	13.5 (8.3)	12.9 (7.3)	−15.1
For diarrheal diseases	0.70 (1.88)	0.64 (2.51)	0.52 (1.72)	0.47 (1.39)	0.31 (1.42)	0.15 (0.45)	−78.6
For malnutrition	0.26 (0.77)	0.25 (0.81)	0.19 (0.57)	0.19 (0.78)	0.16 (0.56)	0.30 (0.79)	15.4
For lower respiratory infections	2.32 (3.27)	2.10 (3.16)	2.00 (3.48)	1.81 (2.81)	1.41 (2.30)	1.46 (2.43)	−37.1
For external causes	0.58 (1.30)	0.40 (1.24)	0.47 (1.07)	0.47 (1.22)	0.45 (1.09)	0.41 (1.03)	−29.3
Hospital rate for children younger than 5 years (per 1000 livebirths)							
Overall	133.7 (92.3)	152.4 (94.3)	132.8 (84.5)	146.9 (95.0)	146.6 (104.6)	143.1 (86.8)	7.0
For diarrheal diseases	57.0 (39.5)	66.4 (46.3)	50.0 (37.3)	54.8 (39.1)	54.5 (43.8)	55.9 (37.9)	−1.9
For malnutrition	2.90 (7.07)	3.01 (4.90)	2.34 (2.99)	2.43 (3.60)	2.13 (4.06)	2.10 (3.03)	−27.6
For lower respiratory infections	73.8 (56.8)	82.9 (59.6)	80.5 (65.0)	89.6 (67.5)	90.0 (69.4)	85.1 (58.9)	15.3
BDH coverage							
BDH coverage of the county population (%)	44.5 (13.1)	42.8 (14.6)	43.1 (14.9)	42.2 (14.8)	35.9 (14.1)	16.7 (10.6)	−62.5
BDH coverage of eligible population (poor households) in the county (%)	63.9 (10.6)	61.1 (12.3)	61.8 (12.2)	60.7 (12.0)	51.5 (12.2)	23.1 (11.1)	−63.8
Determinants of child mortality							
Income per person (monthly, in USD)	266 (361)	273 (405)	350 (911)	364 (1015)	379 (1135)	378 (1001)	42.1
Proportion of households with inadequate sanitation (%)	65.3 (16.5)	64.5 (16.6)	63.7 (16.8)	63.0 (16.9)	62.3 (17.1)	61.6 (17.2)	−5.7
Rate of individuals older than 15 years who are illiterate	9.95 (4.79)	9.70 (4.70)	9.47 (4.62)	9.24 (4.55)	9.02 (4.48)	8.80 (4.41)	−11.6
Total fertility rate	2.42 (0.47)	2.36 (0.46)	2.30 (0.46)	2.24 (0.45)	2.18 (0.46)	2.13 (0.46)	−12.0
Hospital bed rate (per 1000 inhabitants)	0.78 (0.96)	0.81 (0.97)	0.82 (0.98)	0.74 (0.83)	0.75 (0.85)	0.80 (0.90)	2.6
Hospitalization rate (per 1000 inhabitants)^a^	57.0 (24.9)	58.9 (25.2)	59.7 (23.9)	61.7 (26.4)	60.9 (25.1)	63.3 (23.0)	11.1
Physicians rate (per 10,000 inhabitants)	8.68 (4.46)	9.37 (4.79)	9.93 (5.29)	11.10 (6.27)	12.05 (6.52)	13.25 (8.75)	52.6

Table notes: Data are mean (SD). Causes of death were defined according to the International Classification of Diseases, 10th revision: diarrheal diseases (A00, A01, A03, A04, A06–09), malnutrition (E40–46), lower respiratory infections (J10–18, J20–22), and external causes (V01–98)

^a^Hospitalization rate was calculated as the number of hospital discharges for all ages and all causes of one county divided by the total population of the same county and multiplied by 1000. BDH=*Bono de Desarrollo Humano*

Table 2 shows the crude and adjusted associations between BDH coverage (eligible and county population) and under-5 mortality rate. In the analysis, both measures of BDH coverage did not show a statistically significant association with decreasing under-5 mortality rate, even after the adjustment for socio-economic covariates.

**Table 2:** Fixed-effect negative binomial models for association between *Bono de Desarrollo Humano* (BDH) coverage and under-5 mortality rate, 2009–2014, Ecuador

	Counties with intermediate and high quality criterion			Counties with high quality criterion		
	RR crude (95% CI)	RR adjusted (95% CI)		RR crude (95% CI)	RR adjusted (95% CI)	
BDH coverage on eligible population	1.002 (1.001–1.004)	1.000 (0.998–1.002)		1.003 (1.001–1.004)	0.999 (0.997–1.002)	
BDH coverage on county population	1.004 (1.002–1.006)		1.000 (0.997–1.003)	1.005 (1.002–1.008)		0.998 (0.993–1.002)
Hospitalization rate (per 1000 inhabitants)		1.001 (0.998–1.004)	1.000 (0.998–1.004)		1.001 (0.997–1.006)	1.001 (0.997–1.006)
Income per person (monthly, in USD)		1.000 (0.999–1.000)	1.000 (0.999–1.000)		1.000 (1.000–1.001)	1.001 (1.000–1.001)
Proportion of households with inadequate sanitation (%)		1.009 (0.985–1.034)	1.009 (0.985–1.034)		1.010 (0.984–1.038)	1.010 (0.983–1.037)
Rate of individuals older than 15 years who are illiterate		1.023 (0.955–1.096)	1.024 (0.956–1.097)		1.168 (1.027–1.329)	1.170 (1.028–1.332)
Total fertility rate		1.351 (0.932–1.958)	1.353 (0.933–1.962)		0.839 (0.494–1.424)	0.853 (0.502–1.449)
Hospital bed rate (per 1000 inhabitants)		0.992 (0.936–1.053)	0.993 (0.936–1.054)		0.952 (0.875–1.036)	0.949 (0.872–1.033)
Physicians rate (per 10,000 inhabitants)		1.003 (0.993–1.012)	1.003 (0.993–1.012)		0.995 (0.982–1.008)	0.996 (0.983–1.008)
Time (year)		0.992 (0.959–1.026)	0.993 (0.962–1.025)		0.982 (0.942–1.023)	0.977 (0.937–1.019)
Number of observations	864	864	864	432	432	432
Number of counties	144	144	144	72	72	72

Table notes: *RR* Rate Ratio, *CI* Confidence Interval

Table 3 shows adjusted associations between BDH coverage on county population and under-5 mortality rate for some relevant group of causes. The strongest effect of the BDH was on under-5 mortality resulting from malnutrition. One percent increase in BDH coverage was associated with a reduction of 2.9% (95% CI 0.953–0.989) on under-5 mortality resulting from malnutrition in counties with intermediate and high quality of vital information. When we selected only the counties with high quality vital statistics, the observed reductions were higher (reduction of 4.8, 95% CI 0.922–0.983). Lower effect was observed for under-5 mortality resulting from lower respiratory infections (reduction of 0.8% (95% CI 0.984–0.999) for counties with intermediate and high quality vital information. BDH had no effect on mortality rate caused by external causes used as a control group. The associations between BDH coverage on eligible population and under-5 mortality rate for some relevant group of causes are shown in Additional file 1.

**Table 3:** Fixed-effect negative binomial models for adjusted associations between *Bono de Desarrollo Humano* (BDH) coverage on county population and under-5 mortality rate for some relevant group of causes, 2009–2014, Ecuador

	Mortality rate caused by diarrheal diseases		Mortality rate caused by malnutrition		Mortality rate caused by lower respiratory infections		Mortality rate caused by external causes	
	Intermediate and high quality criterion	High quality criterion	Intermediate and high quality criterion	High quality criterion	Intermediate and high quality criterion	High quality criterion	Intermediate and high quality criterion	High quality criterion
	RR adjusted (95% CI)	RR adjusted (95% CI)	RR adjusted (95% CI)	RR adjusted (95% CI)	RR adjusted (95% CI)	RR adjusted (95% CI)	RR adjusted (95% CI)	RR adjusted (95% CI)
BDH coverage on county population	1.007 (0.987–1.027)	0.999 (0.971–1.028)	**0.971 (0.953–0.989)**	**0.952 (0.922–0.983)**	**0.992 (0.984–0.999)**	0.991 (0.980–1.003)	0.999 (0.982–1.015)	1.002 (0.982–1.022)
Hospitalization rate (per 1000 inhabitants)	1.009 (0.991–1.027)	0.992 (0.968–1.017)	1.002 (0.976–1.029)	0.991 (0.955–1.028)	0.996 (0.987–1.006)	1.000 (0.988–1.013)	0.9945 (0.977–1.013)	0.992 (0.969–1.016)
Income per person (monthly, in USD)	0.999 (0.999–1.000)	1.002 (0.999–1.004)	1.000 (0.999–1.001)	0.998 (0.992–1.005)	1.000 (0.999–1.001)	1.000 (0.999–1.002)	1.000 (0.999–1.000)	0.999 (0.998–1.001)
Proportion of households with inadequate sanitation (%)	1.013 (0.953–1.062)	1.044 (0.965–1.130)	1.085 (0.950–1.240)	0.965 (0.827–1.126)	1.050 (0.995–1.108)	1.052 (0.996–1.110)	1.034 (0.954–1.120)	1.083 (0.990–1.184)
Rate of individuals older than 15 years who are illiterate	1.042 (0.890–1.219)	1.031 (0.737–1.443)	0.858 (0.561–1.312)	0.940 (0.526–1.678)	1.106 (0.896–1.366)	1.499 (0.993–2.263)	0.833 (0.684–1.015)	0.574 (0.357–0.923)
Total fertility rate	1.122 (0.206–6.112)	0.445 (0.037–5.340)	7.802 (0.663–91.795)	4.385 (0.120–159.713)	0.851 (0.283–2.560)	0.312 (0.058–1.675)	0.334 (0.045–2.479)	2.298 (0.168–31.507)
Hospital bed rate (per 1000 inhabitants)	1.120 (0.815–1.538)	1.305 (0.838–2.032)	0.857 (0.498–1.473)	0.582 (0.271–1.251)	0.968 (0.818–1.145)	0.798 (0.636–1.002)	1.096 (0.796–1.509)	0.998 (0.637–1.565)
Physicians rate (per 10,000 inhabitants)	0.959 (0.898–1.023)	0.990 (0.910–1.076)	0.926 (0.862–0.994)	0.983 (0.896–1.079)	1.012 (0.984–1.041)	1.001 (0.963–1.041)	0.970 (0.923–1.020)	0.958 (0.903–1.017)
Time (year)	0.880 (0.776–0.998)	0.824 (0.697–0.973)	0.940 (0.932–0.990)	0.952 (0.922–0.983)	0.918 (0.837–1.008)	0.991 (0.980–1.003)	0.9080.795–1.039)	0.995 (0.989–1.005)
Number of observations	552	300	468	258	792	408	624	360
Number of counties	92	50	78	43	132	68	104	60

The bold numbers represent the main effects that are statistically significant

Table notes: *RR* Rate Ratio, *CI* Confidence Interval

Table 4 shows adjusted associations between BDH coverage on county population and under-5 hospitalization rates. One percent increase in BDH coverage was associated with a reduction of 0.2% (95% CI 0.996–0.999) in the overall rate of under-5 hospitalization rates in selected counties with intermediate and high quality vital statistics. When we analyzed only counties with high quality vital information, the effect of BDH was not observed. In addition, BDH coverage reduced hospitalization rates resulting from diarrheal diseases (reduction of 0.6, 95% CI 0.991–0.997) in counties with intermediate and high vital information. This effect remained when we selected only counties with a high quality of vital information (reduction of 0.5, 95% CI 0.990–0.999). The associations between BDH coverage on eligible population and under-5 hospitalization rates for some relevant group of causes are shown in Additional file 2.

**Table 4:** Fixed-effect negative binomial models for adjusted associations between *Bono de Desarrollo Humano* (BDH) coverage and under-5 hospitalization rates, 2009–2014, Ecuador

	Under-5 hospitalization rate		Under-5 hospitalization rate for diarrheal diseases		Under-5 hospitalization rate for malnutrition		Under-5 hospitalization rate for lower respiratory infections	
	Intermediate and high quality criterion	High quality criterion	Intermediate and high quality criterion	High quality criterion	Intermediate and high quality criterion	High quality criterion	Intermediate and high quality criterion	High quality criterion
	RR adjusted (95% CI)	RR adjusted (95% CI)	RR adjusted (95% CI)	RR adjusted (95% CI)	RR adjusted (95% CI)	RR adjusted (95% CI)	RR adjusted (95% CI)	RR adjusted (95% CI)
BDH coverage on county population	**0.998 (0.996–0.999)**	0.999 (0.996–1.003)	**0.994 (0.991–0.997)**	**0.995 (0.990–0.999)**	0.999 (0.991–1.007)	1.005 (0.991–1.019)	1.002 (1.000–1.005)	1.002 (0.998–1.006)
Hospitalization rate (per 1000 inhabitants)	1.017 (1.015–1.020)	1.016 (1.012–1.020)	1.019 (1.016–1.022)	1.020 (1.016–1.025)	1.014 (1.006–1.023)	1.014 (1.002–1.026)	1.015 (1.012–1.028)	1.011 (1.006–1.015)
Income monthly per person in USD	0.999 (0.999–1.000)	0.999 (0.999–1.000)	0.999 (0.999–1.000)	0.999 (0.999–1.000)	0.999 (0.999–1.000)	0.999 (0.998–1.001)	0.999 (0.999–1.000)	1.000 (1.000–1.001)
Proportion of households with inadequate sanitation (%)	1.029 (1.022–1.036)	1.033 (1.022–1.043)	1.033 (1.025–1.042)	1.032 (1.020–1.045)	1.056 (1.030–1.083)	1.056 (1.017–1.096)	1.020 (1.012–1.028)	1.023 (1.012–1.035)
Rate of individuals older than 15 years who are illiterate	1.059 (1.026–1.092)	1.108 (1.053–1.167)	1.112 (1.064–1.162)	1.135 (1.060–1.215)	1.049 (0.948–1.161)	0.980 (0.866–1.110)	1.042 (1.008–1.078)	1.102 (1.042–1.166)
Total fertility rate	0.812 (0.683–0.966)	0.839 (0.613–1.147)	0.875 (0.688–1.113)	0.783 (0.540–1.133)	1.099 (0.554–2.182)	0.848 (0.276–2.607)	1.074 (0.850–1.357)	0.911 (0.630–1.317)
Hospital bed rate (per 1000 inhabitants)	0.938 (0.889–0.990)	0.957 (0.880–1.042)	0.894 (0.832–0.960)	0.878 (0.792–0.972)	0.841 (0.714–0.992)	0.864 (0.680–1.010)	0.955 (0.900–1.013)	1.004 (0.914–1.103)
Physicians rate (per 10,000 inhabitants)	1.000 (0.994–1.007)	0.990 (0.979–1.001)	0.997 (0.988–1.007)	0.989 (0.975–1.004)	1.001 (0.979–1.024)	0.999 (0.960–1.039)	0.994 (0.986–1.002)	0.987 (0.975–0.999)
Time (year)	1.023 (1.005–1.041)	1.036 (1.008–1.064)	0.970 (0.947–0.994)	0.979 (0.945–1.014)	0.985 (0.930–1.042)	0.979 (0.894–1.0171)	1.0711.052–1.091)	1.087 (1.054–1.121)
Number of observations	864	426	870	432	798	396	870	432
Number of counties	144	71	145	72	133	66	145	72

The bold numbers represent the main effects that are statistically significant

Table notes: *RR* Rate Ratio, *CI* Confidence Interval

## Discussion

Our results show that the implementation of the BDH from 2009 to 2014 was associated with a reduction in U5MR from poverty-related causes such as malnutrition and lower respiratory infections at the county level. The effect remained statistically significant after we controlled for all relevant social and economic determinants. BDH also reduced rates of under-5 hospitalizations, overall and from diarrheal diseases.

There is consistent evidence that CCTP had a positive impact on child health and nutrition outcomes, especially among the most vulnerable children. These programs have been effective in increasing the use of preventive services, consumption of healthy foods, immunization rates, and encouraging healthy behaviors [11, 13, 28].

The findings of our study are consistent with other studies that have reported an important positive impact of CCTP on childhood mortality. The Mexican program (*Progresa*) [15] and Brazilian program (*Bolsa Familia*) [22], were able to reduce infant mortality. CCTP in Bolivia increased the survival rates of birth cohorts exposed to the program by 3.5 to 16.8% [29]. In addition, a national Brazilian study showed that *Bolsa Familia* contributed substantially to the reduction of under-5 mortality rates, particularly of poverty-related causes such as malnutrition and diarrhea [14]. Our study, using a similar approach to the Brazilian study, also showed that BDH program significantly reduced under-5 mortality resulting from malnutrition and lower respiratory infections; although in Ecuador there is not a systematic process of verification of compliance with conditions such as in Brazil.

The risk of mortality from diarrhea, pneumonia and malaria are increased greatly in malnourished children, particularly those with severe acute malnutrition [30]. The contribution of CCTPs to the reduction of child undernutrition has been shown in some studies. The Colombian program *Familias en Acción*, was found to have improved the nutritional status of newborns and infants but only for those less than 2 years [31]. The Mexican program, *Progresa* was associated with a better nutritional status and greater growth of children [32, 33]. In Nicaragua, the program was found to have significantly reduced the proportion of underweight and stunted children among the beneficiaries [34]. No significant effects on height and height-for-age z-scores were observed in program from Brazil [35] and Ecuador [18], respectively. In addition, Buser, et al. (2014) [17] showed that in Ecuador, 2 years after families lost the BDH cash transfer, which they had received for 7 years, their young children weighed less, were shorter and were more likely to be stunted than young children in families that kept the cash transfer. Research has already shown that poor families enrolled in CCTP increased food expenditures and improved food security in their households [36]. Families that benefitted from CCTPs reported increased consumption of cereals, meat, and dairy in studies from Brazil [37] and Kenya [28].

Although our results showed that the BDH program has a positive effect on under-5 mortality resulting from malnutrition, we did not obtain the same effect on under-5 mortality resulting from diarrhea. CCTPs are considered powerful child nutrition-sensitive interventions as they address the underlying causes of undernutrition and can enhance the effectiveness of nutrition-specific interventions [38]. However, CCT programs could have minor impact on the reduction of diarrhea outcomes since they may be more sensitive to interventions related to sanitation and hygiene.

Conditional cash transfer programs could affect survival through two main mechanisms. 1) CCT interventions lead to an increase in use of preventive health services among the poor who are underutilizing them, including prenatal care, postnatal care, health and nutrition educational activities for mothers, vaccination schedule, checkups and growth monitoring visits for children younger than 7 years [22, 34, 35, 39]. However, the benefits of improved access may be limited by the quality of existing services [36] and 2) CCT allows the household to improve health-related purchases including higher quality foods, medicines or household materials and equipment that could reduce exposure to infections [14, 36].

Given the important relation between CCTP and the utilization of health and educational facilities, policy makers should assess the adequacy and quality of the existing health and educational infrastructure. This is because meeting the requirements of these programs rely on the availability of basic health services and schools to meet the increased demand created by the programs.

CCTPs appear to decrease the incidence and prevalence of severe illness [36]. The Mexican program showed a 58% reduction in hospital visits for children 0 to 2 years old [39]. We found an effect of CCTP on rates of under-5 hospital rates, overall and for diarrhea. This fact could be explained by two mechanisms: 1) greater and opportune use of preventive care and higher levels of health knowledge may lead to reduce severe cases of illness that needing hospitalization and 2) decreasing the incidence of the diseases by affecting social determinants of health [36].

From 2008 to 2012, the BDH coverage has stood between 60 and 70% in quintile Q1 and 50% in quintile Q2 [5]. It has not been possible to reach higher coverage due to targeting accuracy and the inefficient process for updating information [5]. The Social Registry, created in 2009, obtains mainly its information through household surveys in specific districts of each county, which are selected based on their high poverty rates. Then, this information is used to determine the household’s eligibility for benefits [4]. Probably, this way of data collection has resulted in the exclusion of some specific population group that being in conditions of extreme poverty and poverty are not part of the system. By 2014, the coverage of BDH program fell drastically because of fiscal constraints [5]. The decline in oil prices in 2014 made evident the macroeconomic vulnerabilities of Ecuador, which resulted in a decrease in public spending, including spending on social assistance [5]. Poverty reduction then stagnated as well as economic growth. The contribution of public transfers to poverty reduction decreased between 2014 and 2017, and employment and private transfers became the main drivers of poverty reduction [5]. In fact, stricter eligibility conditions were introduced to the BDH program in 2014 so that only people in extreme poverty could receive the monetary transfer [5]. This change allowed the exit of more than 600.000 beneficiaries, but it did not contemplate a process to incorporate potential new beneficiaries [6]. As a result, the coverage rates dropped substantially but the targeting improved as the number of beneficiaries of the first quintile increased [5]. The implementation of BDH exit strategies is required, which includes linking the beneficiaries who are not extremely poor to productive inclusion programs [5].

Our study’s limitations include the ecological design and the use of the county as our unit of analysis due to data availability. In addition, we selected only counties with intermediate and high quality of vital statistics in order to improve the internal validity of our study. We observed that counties with adequate quality of vital statistics showed better socioeconomic indicators than excluded counties. Therefore, the selection of counties with vital information of adequate quality might limit the generalizability of the results to the all country. While the selection of counties with only high quality vital information since the initial year of the study would contribute to unbiased BDH effect estimates, its consequential reduction in number of observations would also reduce the statistical power of the study. On the other hand, the use of more relaxed criteria (intermediate and high quality) increases the number of observations but could introduce negative bias (reduction of effect) in BDH impact estimates, as already discussed elsewhere [14]. We decided to include both cases in the tables to show these effects empirically.

Another potential limitation of our study is that we were not able to test the difference between the pre-intervention (before 2003) and post-intervention trends, due to the limited availability of adequate quality data in the period before the implementation of the intervention. However, the differences in pre-intervention trends are also explained and adjusted by the observed variables in the models.

Finally, the linear interpolation and extrapolation of some covariates from decennial census could introduce bias. However, these estimates do not affect the results because slightly fluctuations in some structural determinants are expected during the study period.

## Conclusions

CCTPs like *Bono de Desarrollo Humano* have great potential to improve the population health of the poorest populations. The BDH program had a positive effect on under-5 mortality resulting from malnutrition and lower respiratory infections and on under-5 hospital rates. These results are the reflection of a successful government social policy that guarantee a minimum level of consumption for families and to reduce chronic malnutrition and preventable childhood diseases. Conditional requirements should be effectively communicated, controlled and enforced with the support of an effective Primary Health System to achieve a greater effect on health outcomes.

The last revision of Social Registry and adjustment of the eligibility criteria was performed in 2014. Therefore, a large number of families were excluded from the BDH program. The coverage should be maintained -or increased in a period of economic crisis- and its implementation strengthened. In addition, these changes will require careful monitoring and evaluation of the impact of the program to support its effectiveness in the reduction of poverty and health improvements.

## Additional files

Additional file 1: Fixed-effect negative binomial models for adjusted associations between *Bono de Desarrollo Humano* (BDH) coverage on eligible population and under-5 mortality rate for some relevant group of causes, 2009–2014, Ecuador. (DOCX 20 kb)

Additional file 2: Fixed-effect negative binomial models for adjusted associations between *Bono de Desarrollo Humano* (BDH) coverage on eligible population and under-5 hospitalization rates, 2009–2014, Ecuador. (DOCX 21 kb)

## Data Availability

Data are with the authors (Ana L. Moncayo and Davide Rasella) and available for sharing upon request. The datasets supporting the conclusions of this article are available from public websites hosted by Ecuadorian government agencies. Birth and death data, hospitalizations, health system resources, population estimates, illiteracy and sanitation were obtained from https://www.ecuadorencifras.gob.ec/banco-de-informacion/. Population projections were obtained from https://www.ecuadorencifras.gob.ec/proyecciones-poblacionales/. BDH coverage can be obtained from http://www.conocimientosocial.gob.ec/pages/ProgramasSociales/herramientasProgramas.jsf. Data on income was available from https://www.bce.fin.ec/index.php/component/k2/item/763.

## Abbreviations

BDH: Bono de Desarrollo Humano
CCTP: Conditional Cash Transfer Programs
CI: Confidence Interval
CISeAL: Centro de Investigación para la Salud en América Latina
CP: County Population
EP: Eligible population
ICD: International Classification of Diseases
LAC: Latin American and the Caribbean
MDG 4: Millennium Development Goal 4
MDG: Millennium Development Goal
PUCE: Pontificia Universidad Católica del Ecuador
RR: Rate Ratio
U5MR: Under-five mortality rate
