# Supplementary material for: Strong effect of Ecuador’s conditional cash transfer program on childhood mortality from poverty-related diseases: a nationwide analysis
Source: BMC Public Health. 2019 Aug 17;19:1132. doi: 10.1186/s12889-019-7457-y (PMC6697994; doi:10.1186/s12889-019-7457-y)
Supplement: Supplementary file 1 — Fixed-effect negative binomial models for adjusted associations between Bono de Desarrollo Humano (BDH) coverage on eligible population and under-5 mortality rate for some relevant group of causes, 2009–2014, Ecuador. (DOCX 20 kb) [file 12889_2019_7457_MOESM1_ESM.docx]

| **Table S1. Fixed-effect negative binomial models for adjusted associations between *Bono de Desarrollo Humano* (BDH) coverage on eligible population and under-5 mortality rate for some relevant group of causes, 2009-2014, Ecuador.** | | | | | | | | | | | |
| --- | --- | --- | --- | --- | --- | --- | --- | --- | --- | --- | --- |
|  | **Mortality rate caused by diarrheal diseases** | |  | **Mortality rate caused by malnutrition** | |  | **Mortality rate caused by lower respiratory infections** | |  | **Mortality rate caused by external causes** | |
|  | **Intermediate and high quality criterion** | **High quality criterion** |  | **Intermediate and high quality criterion** | **High quality criterion** |  | **Intermediate and high quality criterion** | **High quality criterion** |  | **Intermediate and high quality criterion** | **High quality criterion** |
|  | **RR adjusted**  **(95% CI)** | **RR adjusted (95% CI)** |  | **RR adjusted**  **(95% CI)** | **RR adjusted (95% CI)** |  | **RR adjusted**  **(95% CI)** | **RR adjusted**  **(95% CI)** |  | **RR adjusted**  **(95% CI)** | **RR adjusted (95% CI)** |
| **BDH coverage on eligible population** | 1.005  (0.992-1.018) | 1.000  (0.983-1.017) |  | **0.985**  **(0.973-0.998)** | **0.971**  **(0.954-0.989)** |  | **0.995**  **(0.989-0.999)** | 0.994  (0.987-1.002) |  | 0.999  (0.985-1.009) | 0.994  (0.982-1.007) |
| Hospitalization rate (per 1000 inhabitants) | 1.009  (0.990-1.027) | 0.992  (0.968-1.017) |  | 1.003  (0.977-1.030) | 0.990  (0.956-1.027) |  | 0.997  (0.988-1.006) | 1.001  (0.988-1.014) |  | 0.995  (0.977-1.014) | 0.992  (0.968-1.016) |
| Income monthly per person in USD | 0.999  (0.999-1.000) | 1.002  (0.999-1.004) |  | 0.999  (0.999-1.001) | 0.997  (0.993-1.000) |  | 1.000  (0.999-1.001) | 1.000  (0.999-1.002) |  | 1.000  (0.999-1.000) | 1,001  (0.999-1.002) |
| Proportion of households with inadequate sanitation | 1.014  (0.960-1.072) | 1.044  (0.966-1.129) |  | 1.096  (0.956-1.257) | 0.955  (0.844-1.080) |  | 1.047  (0.989-1.109) | 1.049  (0.994-1.107) |  | 1.034  (0.954-1.120) | 1.085  (0.997-1.180) |
| Rate of individuals older than 15 years who are illiterate | 1.044  (0.892-1.223) | 1.029  (0.738-1.437) |  | 0.841  (0.559-1.265) | 0.9001 (0.610-1.330) |  | 1.090  (0.882-1.348) | 1.471  (0.971-2.230) |  | 0.832  (0.684-1.013) | 0.495  (0.301-0.816) |
| Total fertility rate | 1.184  (0.216-6.499) | 0.441  (0.037-5.290) |  | 5.334  (0.489-58.165) | 2.833  (0.082-98.351) |  | 0.795  (0.264-2.397) | 0.306  (0.057-1.648) |  | 0.326  (0.045-2.369) | 0.384  (0.017-8.412) |
| Hospital bed rate (per 1000 inhabitants) | 1.123  (0.816-1.545) | 1.307  (0.840-2.034) |  | 0.867  (0.509-1.478) | 0.610  (0.285-1.304) |  | 0.971  (0.822-1.147) | 0.802  (0.639-1.006) |  | 1.072  (0.776-1.482) | 0.873  (0.546-1.394) |
| Physicians rate (per 10000 inhabitants) | 0.958  (0.898-1.023) | 0.989  (0.910-1.076) |  | 0.927  (0.861-0.999) | 0.987  (0.899-1.082) |  | 1.012  (0.984-1.042) | 1.001  (0.963-1.041) |  | 0.970  (0.923-1.019) | 0.991  (0.931-1.054) |
| Time (year) | 0.886  (0.775-1.014) | 0.826  (0.694-0.985) |  | 0.825  (0.680-0.983) | 0.811  (0.628.1.047) |  | 0.918  (0.837-1.008) | 0.930  (0.837-1.034) |  | 0.905  (0.784-1.046) | 0.804  (0.678-0.952) |
| Number of observations | 552 | 300 |  | 468 | 258 |  | 792 | 408 |  | 624 | 360 |
| Number of counties | 92 | 50 |  | 78 | 43 |  | 132 | 68 |  | 104 | 60 |
| Table notes: RR = rate ratio; CI = Confidence Interval.  The bold numbers represent the main effects that are statistically significant. | | | | | | | |  |  |  |  |
