# Supplementary material for: Strong effect of Ecuador’s conditional cash transfer program on childhood mortality from poverty-related diseases: a nationwide analysis
Source: BMC Public Health. 2019 Aug 17;19:1132. doi: 10.1186/s12889-019-7457-y (PMC6697994; doi:10.1186/s12889-019-7457-y)
Supplement: Supplementary file 2 — Fixed-effect negative binomial models for adjusted associations between Bono de Desarrollo Humano (BDH) coverage on eligible population and under-5 hospitalization rates, 2009–2014, Ecuador. (DOCX 21 kb) [file 12889_2019_7457_MOESM2_ESM.docx]

| **Table S2. Fixed-effect negative binomial models for adjusted associations between *Bono de Desarrollo Humano* (BDH) coverage on eligible population and under-5 hospitalization rates, 2009-2014, Ecuador.** | | | | | | | | | | | |  |
| --- | --- | --- | --- | --- | --- | --- | --- | --- | --- | --- | --- | --- |
|  | **Under-5 hospitalization**  **rate** | |  | **Under-5 hospitalization rate for diarrheal diseases** | |  | **Under-5 hospitalization rate for malnutrition** | |  | **Under-5 hospitalization rate for lower respiratory infections** | | |
|  | **Intermediate and high quality criterion** | |  | **Intermediate and high quality criterion** | |  | **Intermediate and high quality criterion** | |  | **Intermediate and high quality criterion** | |  |
|  | **RR adjusted**  **(95% CI)** | **RR adjusted**  **(95% CI)** |  | **RR adjusted**  **(95% CI)** | **RR adjusted**  **(95% CI)** |  | **RR adjusted**  **(95% CI)** | **RR adjusted**  **(95% CI)** |  | **RR adjusted**  **(95% CI)** | **RR adjusted**  **(95% CI)** |  |
| **BDH coverage on eligible population** | **0.999**  **(0.998-0.999)** | 0.999  (0.997-1.002) |  | **0.996**  **(0.994-0.998)** | **0.996**  **(0.994-0.999)** |  | 0.999  (0.994-1.005) | 1.002  (0.994-1.010) |  | 1.002  (1.001-1.004) | 1.002  (0.999-1.004) |  |
| Hospitalization rate (per 1000 inhabitants) | 1.017  (1.015-1.020) | 1.016  (1.012-1.020) |  | 1.019  (1.016-1.022) | 1.021  (1.016-1.026) |  | 1.014  (1.006-1.022) | 1.014  (1.003-1.026) |  | 1.014  (1.011-1.017) | 1.011  (1.006-1.015) |  |
| Income monthly per person in USD | 0.999  (0.999-1.000) | 1.000  (0.999-1.000) |  | 0.999  (0.999-1.000) | 0.999  (0.999-1.000) |  | 0.999  (0.999-1.000) | 0.999  (0.998-1.001) |  | 1.000  (0.999-1.000) | 1.000  (0.999-1.000) |  |
| Proportion of households with inadequate sanitation (%) | 1.029  (1.022-1.036) | 1.032  (1.022-1.043) |  | 1.032  (1.023-1.040) | 1.030  (1.017-1.042) |  | 1.056  (1.030-1.082) | 1.058  (1.021-1.096) |  | 1.021  (1.013-1.029) | 1.024  (1.012-1.036) |  |
| Rate of individuals older than 15 years who are illiterate | 1.059  (1.026-1.093) | 1.108  (1.052-1.166) |  | 1.109  (1.061-1.159) | 1.132  (1.058-1.212) |  | 1.049  (0.948-1.161) | 0.980  (0.866-1.109) |  | 1.043  (1.008-1.079) | 1.103  (1.043-1.167) |  |
| Total fertility rate | 0.809  (0.681-0.960) | 0.836  (0.610-1.145) |  | 0.838  (0.657-1.069) | 0.756  (0.520-1.098) |  | 1.091  (0.550-2.166) | 0.879  (0.285-2.709) |  | 1.106  (0.877-1.395) | 0.927  (0.641-1.340) |  |
| Hospital bed rate (per 1000 inhabitants) | 0.940  (0.891-0.992) | 0.946  (0.871-1.0128) |  | 0.897  (0.835-0.963) | 0.882  (0.798-0.976) |  | 0.843  (0.715-0.994) | 0.858  (0.675-1.090) |  | 0.955  (0.901-1.013) | 1.004  (0.915-1.102) |  |
| Physicians rate (per 10000 inhabitants) | 1.000  (0.994-1.007) | 0.959  (0.882-1.043) |  | 0.997  (0.988-1.006) | 0.989  (0.975-1.003) |  | 1.001  (0.979-1.024) | 0.999  (0.960-1.039) |  | 0.994  (0.986-1.002) | 0.987  (0.975-0.999) |  |
| Time (year) | 1.024  (1.005-1.043) | 1.036  (1.008-1.066) |  | 0.966  (0.942-0.991) | 0.976  (0.941-1.012) |  | 0.988  (0.929-1.050) | 0.974  (0.889-1.068) |  | 1.078  (1.057-1.100) | 1.092  (1.058-1.126) |  |
| Number of observations | 864 | 426 |  | 870 | 432 |  | 798 | 396 |  | 870 | 432 |  |
| Number of counties | 144 | 71 |  | 145 | 72 |  | 133 | 66 |  | 145 | 72 |  |
| Table notes: RR= Rate Ratio, CI= confidence interval  The bold numbers represent the main effects that are statistically significant. | | |  |  |  |  |  |  |  |  |  |  |
